# From the Editor’s Desk

**Published:** 2017

**Authors:** Pat Commerford

**Affiliations:** Editor-in-Chief

The gap between clinical practice guidelines and usual clinical practice is acknowledged to exist worldwide, is seldom measured, but is considered to be important. In this issue, Ale and Braimoh (page 72) report on the knowledge of hypertension guidelines in a large sample of primary care physicians in Nigeria. Deficiencies in knowledge seemed to translate to deficiencies in diagnosis and management.

In a cross-sectional study, Ononamadu and colleagues (page 92) compared the performance of eight anthropometric indices of obesity: body mass index (BMI), ponderal index, waist circumference (WC), hip circumference, waist–hip ratio, waist–height ratio, body adiposity index and conicity index as correlates and potential predictors of risk for hypertension and prehypertension in a Nigerian population, and also the possible effect of combining two or more indices in that regard. In a related but very different study, Onen (page 86) investigated anthropometric data from 215 male and 203 female patients seen in a specialist clinic in Gaborone, Botswana, between 2005 and 2015 to establish appropriate cut-off points for WC corresponding to a BMI of 30 kg/m^2^. Relative risks for cardiometabolic disorders were calculated for different BMIs and WCs. He proposes new cut-off values, which may be useful in studies in other sub-Saharan countries.

The importance of studies such as those above is emphasised by the report from Mwita and co-authors (page 112) on the clinical characteristics and outcomes of patients admitted to hospital in Botswana with acute heart failure. The patients were younger than in similar series in other parts of the world and had a high short-term mortality rate. Components of the metabolic syndrome were considered to be important in causation. Attempts to curb the mortality associated with the metabolic syndrome require ongoing efforts to identify it and treat its components in Africa.

